# Supplementary material for: Efficacy of Jackfruit365™ Green Jackfruit Flour Fortified Diet on Pegfilgrastim to Prevent Chemotherapy-Induced Leukopenia, Irrespective of Tumor Type or Drugs Used—A Retrospective Study
Source: Biomolecules. 2020 Feb 2;10(2):218. doi: 10.3390/biom10020218 (PMC7072368; doi:10.3390/biom10020218)
Supplement: Supplementary file 1 [file biomolecules-10-00218-s001.pdf]

**Table S1: Baseline Table Which Include Tumor Type between Study and Control Group Patients**

| TUMOR TYPE | STUDY GROUP | CONTROL GROUP |
|---|---|---|
| Breast Cancer | 20 | 20 |
| Ovary Cancer | 6 | 5 |
| Tongue Cancer | 5 | 3 |
| Lung Cancer | 3 | 3 |
| Rectum Cancer | 2 | 1 |
| Endometrium Cancer | 2 | 1 |
| Pancreas Cancer | 3 | 1 |
| Oesophagus junction Cancer | 1 | 1 |
| Gastro- Oesophagus Cancer | 2 | 0 |
| Ileocecal Junction Cancer | 1 | 0 |
| Urethra Cancer | 1 | 0 |
| Buccal Mucosa | 1 | 0 |
| Kidney Nephrectomy Cancer | 1 | 0 |
| Sarcomatoid Cancer | 1 | 0 |
| Soft Tissue sarcoma of Left Pelvic | 1 | 0 |
| Leiomyosarcoma uterus | 0 | 3 |
| Olfactory Neuroblastoma | 0 | 1 |
| Soft tissue Sarcoma of rt. Upper limb | 0 | 1 |
| Stomach Cancer | 0 | 3 |
| Soft tissue Sarcoma of Thigh | 0 | 1 |
| Soft tissue Sarcoma of left thigh posteromedial | 0 | 1 |
| Squamoua Cell Ca of right leg | 0 | 1 |

**Table S2: Baseline Table of Drug Type between Study and Control Group Patients**

| DRUG TYPE | STUDY GROUP | CONTROL GROUP |
|---|---|---|
| Albumin Bound Paclitaxel | 32 | 35 |
| Carboplatin | 18 | 16 |
| Liposomal Doxorubicin | 11 | 14 |
| Oxaliplatin | 9 | 8 |
| Herceptin or Biosimilar | 11 | 5 |
| Solvent free Docetaxel | 10 | 5 |
| Bevazizumab | 6 | 8 |
| Nimotuzumab | 7 | 2 |
| Holoxan | 4 | 3 |
| Epirubicin | 2 | 4 |

**Table S3: Analytical Report for Amino Acid Profile and Potassium**

# ANALYTICAL REPORT

| | | | |
|---|---|---|---|
| **Sample code:** | 260-2019-08000117 | **Received on:** | 02.08.2019 |
| **Sample name:** | Jack Fruit 365 Jack of Fibr | **Analysed between:** | 02.08.2019 - 16.08.2019 |
| **Sample reference** | Green Jackfruit Flour<br>Batch number: IPA8G1011<br>Mfg. date: 03/2019<br>Best before: 12 months from mfg. | | |
| **Sample appearance:** | Black colour powder | | |
| **Quantity received:** | 200 g | **Condition on receipt:** | Good |
| **Sample packing:** | Trade pack | **Sampling:** | NOT SAMPLED BY EUROFINS |

| **MINERALS** | | | ***Method*** | ***Result Unit*** |
|---|---|---|---|---|
| **IR093** | **IR** | *Sodium (Na) | AOAC 2011.14 | <0.2 mg/100 g |
| **IR094** | **IR** | *Potassium (K) | AOAC 2011.14 | 1470 mg/100 g |

| **CHEMICAL** | | | ***Method*** | ***Result Unit*** |
|---|---|---|---|---|
| **IR938** | **IR** | *Ferulic acid | Polish J Food and Nutrition Sciences | 9.13 mg/kg |
| **IR939** | **IR** | *p-Coumaric acid | Polish J Food and Nutrition Sciences | 0.15 mg/kg |
| **IR0U9** | **IR** | *Polyphenols calculated as catechine equivalent | By Customer Protocol | <0.01 g/100 g |
| **IR25G** | **IR** | *Gallic acid | ISO 14502-2:2005 | 1.23 mg/kg |

**AMINO-ACID PROFILE** ***Result Unit***

**IR130 IR Amino Acids Method: EASI-CHE-SOP-25, LC-FLD**

| | |
|---|---|
| * Threonine | 0.18 g/100 g |
| * Tyrosine | 0.29 g/100 g |
| * Valine | 0.44 g/100 g |
| * Proline | 0.39 g/100 g |
| * Serine | 0.47 g/100 g |
| * Methionine | 0.08 g/100 g |
| * Aspartic Acid | 1.27 g/100 g |
| * Cystein +Cystine | 0.05 g/100 g |
| * Arginine | 0.50 g/100 g |
| * Histidine | 0.15 g/100 g |
| * Lysine | 0.35 g/100 g |
| * Phenylalanine | 0.43 g/100 g |

The results may not be reproduced except in full, without a written approval of the laboratory. The results relate only to the sample analysed.

Eurofins Analytical Services India Private Limited
Plot no 157, First Floor, Udyog Vihar, Phase 1, Gurugram-122016, Haryana, India
Phone: 124 625 0300, Email: delhifood@eurofins.com, website: www.eurofins.in, CIN: U73100KA2009PTC049992
Redg. Office: #540/1, Doddanakundi Industrial Area 2, Hoodi, Whitefield, Bengaluru-560048, Karnataka, India, Phone: +91 80 30982500



eurofins

**Report code:** AR-19-KV-005098-01

**Batch code:** EUINDE-00007122

**AMINO-ACID PROFILE** ***Result Unit***

**IR130 IR Amino Acids Method: EASI-CHE-SOP-25, LC-FLD**

| | |
|---|---|
| * Leucine | 0.69 g/100 g |
| * Isoleucine | 0.37 g/100 g |
| * Alanine | 0.28 g/100 g |
| * Glycine | 0.81 g/100 g |
| * Glutamic acid | 0.75 g/100 g |
